# Current practices for diagnosis and management of Canine Cognitive Dysfunction Syndrome in the United States

**DOI:** 10.3389/fvets.2025.1685430

**Published:** 2025-10-29

**Authors:** Katherine E. Simon, Margaret E. Gruen, Natasha J. Olby

**Affiliations:** Department of Clinical Sciences, College of Veterinary Medicine, North Carolina State University, Raleigh, NC, United States

**Keywords:** canine cognitive dysfunction syndrome, veterinarian, diagnosis, management, selegiline, canine dementia

## Abstract

**Introduction:**

There are currently no accepted guidelines for the diagnosis and management of Canine Cognitive Dysfunction Syndrome (CCDS). The objective of this study was to describe the current diagnostic and management practices regarding CCDS by veterinarians in the United States (US).

**Methods:**

An anonymous online survey was distributed to veterinary practitioners from January to May 2025. The survey included questions regarding patient population, CCDS diagnosis and treatment and client interactions.

**Results:**

A total of 318 survey responses were obtained from veterinarians who saw companion dogs regularly. Nearly all (97.2%) had made a diagnosis of CCDS in their career, citing patient history and clinical signs/ behavioral changes as the tools they use to make a diagnosis. Most veterinarians (approximately 80%) rarely or never referred their potential CCDS cases to a veterinary specialist. When managing their CCDS patients, pharmaceuticals are most often recommended, specifically selegiline. Selegilline was also considered most effective in managing CCDS, however this view was held by only about 30% of veterinarians. Responses reflected uncertainty regarding best practices and treatment efficacies, with veterinarians citing lack of knowledge and owner-related barriers such as lack of interest or financial constraints as factors which hinder treatment recommendations.

**Discussion:**

Results from this survey underscore there are still significant gaps in knowledge as to best practices for the diagnosis and management of CCDS. Clear CCDS diagnostic and management guidelines are needed to support veterinarians and address the therapeutic needs of patients.

## Introduction

Canine Cognitive Dysfunction Syndrome (CCDS) is a canine analog of Alzheimer’s Disease (AD), characterized by behavioral changes which develop with advanced age (1–3). These include changes in social interactions (reduced responsiveness to familiar people/objects, aversion to petting), loss of spatial orientation (disorientation, wandering), disturbance of sleep–wake cycles, development of house soiling (urination or defecation in an unusual location), and development of anxiety and/or aggression (4, 5). Several studies have characterized AD-like neuropathology in elderly dogs, with findings such as amyloid beta accumulation, oxidative damage and neuroinflammation correlating with performance on cognitive assessments and/or behavioral questionnaires (6–9).

In human medicine, diagnosis of AD has evolved over the last 50 years, now encompassing different stages of disease (preclinical, mild cognitive impairment (MCI) and dementia) (10–12). In 2024, the National Institute on Aging and the Alzheimer’s Association (NIA-AA) released updated guidelines to inform diagnosis and staging of AD (13). These guidelines emphasize a biological definition of disease, relying heavily on biomarkers and diagnostic imaging, including blood-based markers, cerebrospinal fluid and positron emission tomography (PET) to achieve this. Biomarkers can be categorized as core, non-specific, or non-AD, and different types may offer differing utility to diagnosis, staging or prognosis (13). Given that biomarker abnormalities often precede clinical symptoms, they are critical to identifying individuals in the earliest phases of disease (14–16). In addition to biomarkers, accurate diagnosis relies upon clinical and functional evaluation including thorough history, physical examination, cognitive testing, advanced imaging, and laboratory tests (17). However, diagnostic workups are inconsistent due to differing individual presentations/trajectories, clinician preference, cost, or limited access to diagnostic resources (18–20). This inconsistency, even with formally established guidelines, has posed a significant barrier to understanding and treating AD (21, 22).

In dogs, the absence of formal diagnostic criteria for CCDS makes consistent and standardized clinical evaluation even more difficult. The behavioral abnormalities associated with CCDS can be captured using validated caregiver questionnaires (4, 5, 23). However, these are not specific diagnostic tests, and many of the behaviors described can be caused by other medical conditions such as (but not limited to) sensory decline (vision or hearing loss), chronic pain, renal disease, or intracranial neoplasia (24, 25). As such, a thorough diagnostic workup including signalment, detailed patient history, physical and neurological examination, laboratory testing and advanced imaging (radiographs, MRI) is indicated to exclude other differential diagnoses (3, 24, 26). While a few studies have suggested circulating biomarkers to be indicative of CCDS based on either caregiver questionnaires or cognitive assessments, they are not yet considered to be diagnostic (7, 27–31). As such, they have yet to employed in a point-of-care setting (32).

Diagnostic challenges are compounded by limited agreement on best therapeutic approaches. Currently, selegiline is the only drug in the United States approved by the Food and Drug Administration for treatment of canine cognitive dysfunction (33). However, there are many supplements and specialized diets formulated to promote cognitive improvement in aging dogs (34–38). Despite the availability of these products, the evidence supporting their efficacy remains variable. Further, it can be difficult to compare management options given the varying study designs, cognitive outcomes, and populations in which each was investigated. This makes selecting an appropriate intervention to recommend to patients challenging.

Little research has been performed to explore the perspectives of veterinarians regarding the diagnostics and treatments they perceive are most helpful and effective in clinical practice. The objective of this study was to describe the current practices of veterinarians in the United States (US) when making a diagnosis and management plan in a patient with CCDS. We hoped to identify gaps in practice which may be addressed through intentional educational and research efforts.

## Materials and methods

### Survey development

An anonymous survey was developed on the online survey platform Qualtrics© (Qualtrics, Provo, UT, 2025). The survey asked a series of questions about how veterinarians establish a diagnosis of CCDS, how they treat the condition, and how they learned about it (Supplementary Data Sheet 1). The first section contained information on the respondent’s clinical practice, the next section contained information on the diagnosis of CCDS. The third section was on the treatment of CCDS and the fourth was on interactions with senior pet clients. All questions were multiple choice, though some questions included an “other” response which prompted further written explanation if selected. To ensure relevance and reduce respondent burden, the survey was designed with skip and if/then logic so that questions were only displayed to respondents when applicable (based on their prior responses). All survey logic is described in Supplementary Data Sheet 1. The study was declared exempt through NC State Institutional review board for the use of human subjects in research because all data were collected anonymously.

### Survey distribution

To distribute the survey across the US, we reached out to State and Regional Veterinary Medical Associations (Supplementary Data Sheet 2) and we shared the survey with veterinarians via social media platforms (primarily facilitated by veterinary content creators). Circulation began in January 2025 and ended May 2025. In order to collect data that represented the general population of primary veterinarians, we aimed to gather 370 completed responses. This estimate is consistent with the Qualtrics© sample size calculator^1^; and aligns with the recommendations for populations of 10,000 or larger at a 95% confidence level and 5% margin of error, as outlined by Bartlett et al. (39). We felt this appropriate given the American Veterinary Medical Association reported the population of practicing US veterinarians in 2024 was 130,000, but not all of these regularly see dogs in their patient population.

### Data analysis

Responses were summarized and reported as both fractions and percentages. Only complete responses from currently practicing US veterinarians were included in the study and respondents who did not see dogs in their routine patient population were excluded. A single Chi Square test and contingency table was built to test association between whether the respondent learned about CCDS in veterinary school based on graduation year. A *p* value of <0.05 was considered significant. All statistics and graphical representations were conducted in JMP^®^, Student Version 18.2.0 (SAS Institute Inc., Cary, NC, 2025).

## Results

We obtained 471 survey responses, of which 319 were from veterinarians practicing in the US and completed in entirety. One respondent reported that they did not see dogs in their routine patient population and was therefore excluded from our analysis. Therefore, the final number of survey responses included in the descriptive analysis was 318; however, the number of responses included in subsequent analyses vary based on question-specific inclusion criteria.

### Respondent population

**Figure 1:**
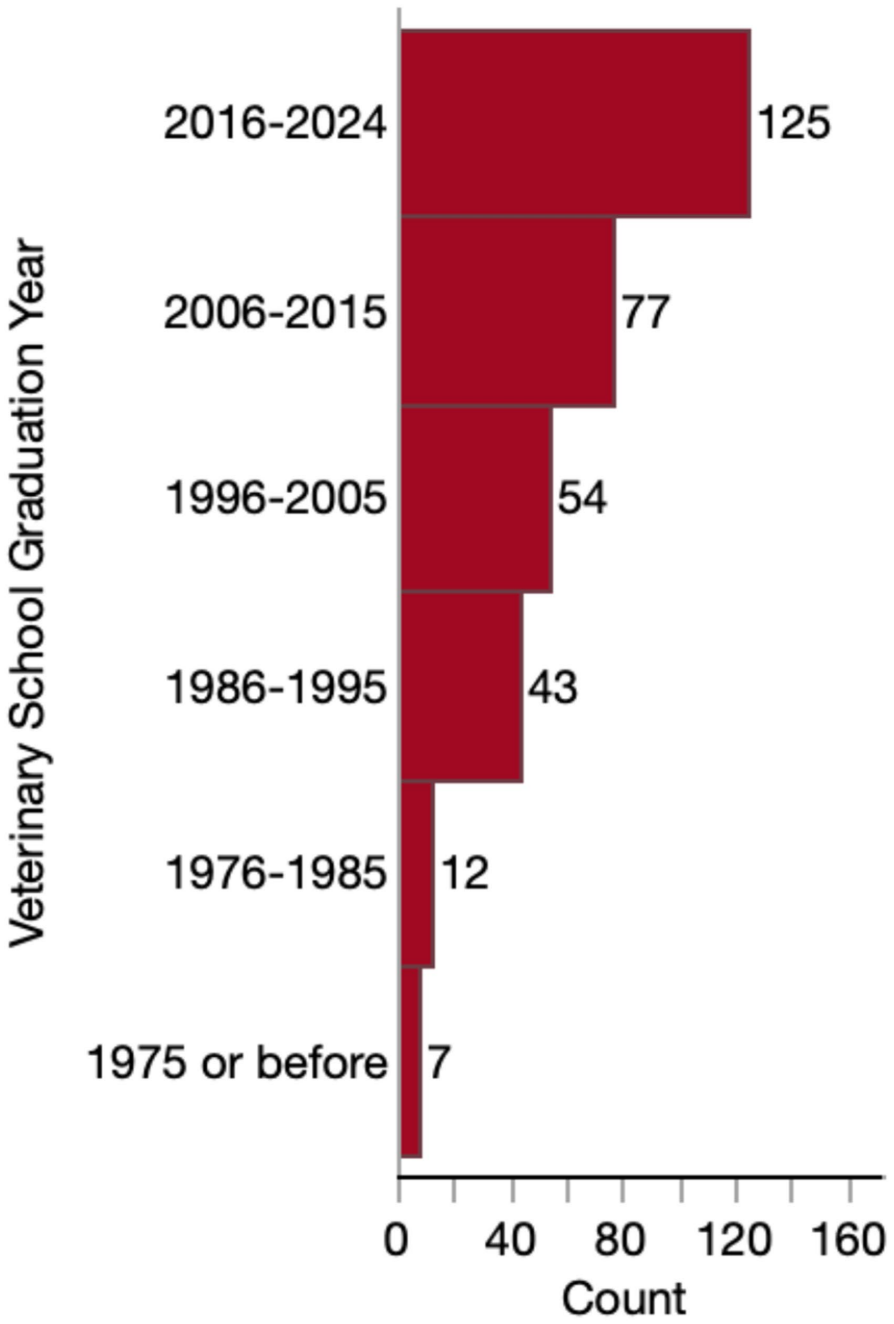
Histogram of survey respondents’ veterinary school graduation year.

Most respondents (279/318, 87.7%) were general practitioners or primary care veterinarians. The remaining respondents consisted of specialists (18/318, 5.7%) (including 7 internists, 3 behaviorists, 3 ophthalmologists, 3 emergency critical care specialists, 1 sports medicine/rehabilitation specialist and 1 surgeon), interns (2/318, 0.6%), residents (2/318, 0.6%) or other (17/318, 5.3%). “Other” respondents included: 8 emergency/urgent care veterinarians, 5 end-of-life care veterinarians, 2 relief veterinarians, 1 rehabilitation veterinarian, and 1 shelter medicine veterinarian. Accordingly, 79.6% (253/318) work in a general practice setting, 6.0% (19/318) work in a specialty practice, 3.8% (12/318) work at an in-home/mobile practice, 0.9% (3/318) work in an emergency clinic or urgent care clinic, and 9.4% (30/318) worked in a combination of these settings. Only one veterinarian reported working exclusively in an academic setting. Most (125/318, 39.3%) of these veterinarians graduated veterinary school between 2016 and 2024, and few respondents graduated before 1986 (Figure 1).

**Figure 2:**
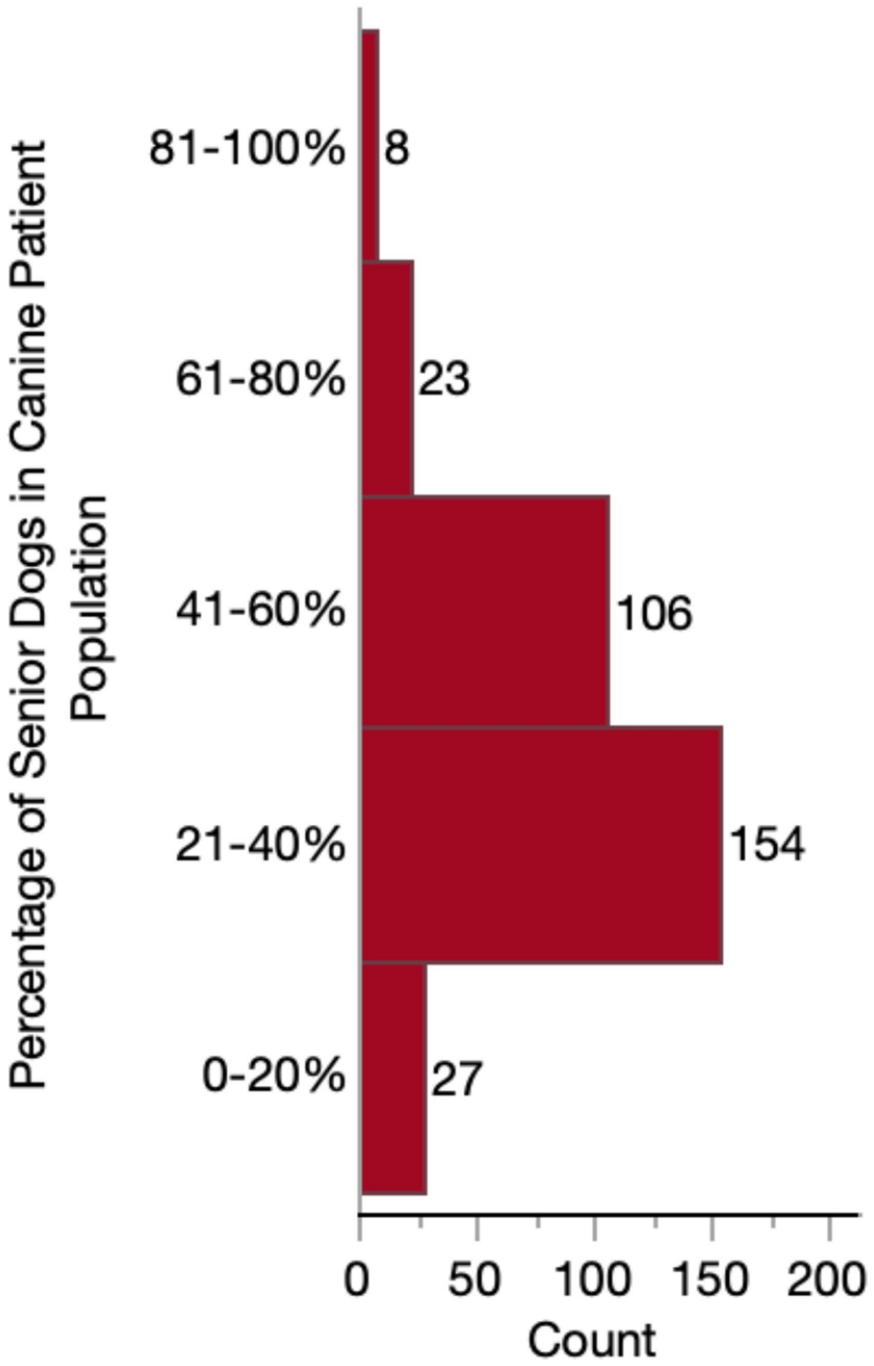
Histogram of percentage of respondents’ canine patients considered to be senior.

Almost half of respondents (149/318, 46.9%) considered themselves to have a special interest in geriatric medicine. Whereas only 64/318 (20.1%) responded “no,” and 105/318 (33.0%) responded that they were “neutral.” The majority (217/318 68.2%) of practitioners reported that their practices do not provide a specialized or distinct senior visit. However, the percentage of senior-aged dogs in their caseload varied substantially, with most (260/318, 81.8%) falling between 21 and 60% of their canine patient population (Figure 2). When considering these senior dogs, most respondents were moderately (145/318, 45.6%) or very (106/318, 33.3%) concerned about CCDS. Far fewer reported having slight (43/318, 13.5%) or extreme (24/318, 7.5%) concern. No veterinarians reported that they were not at all concerned about CCDS in their senior dog patients.

**Figure 3:**
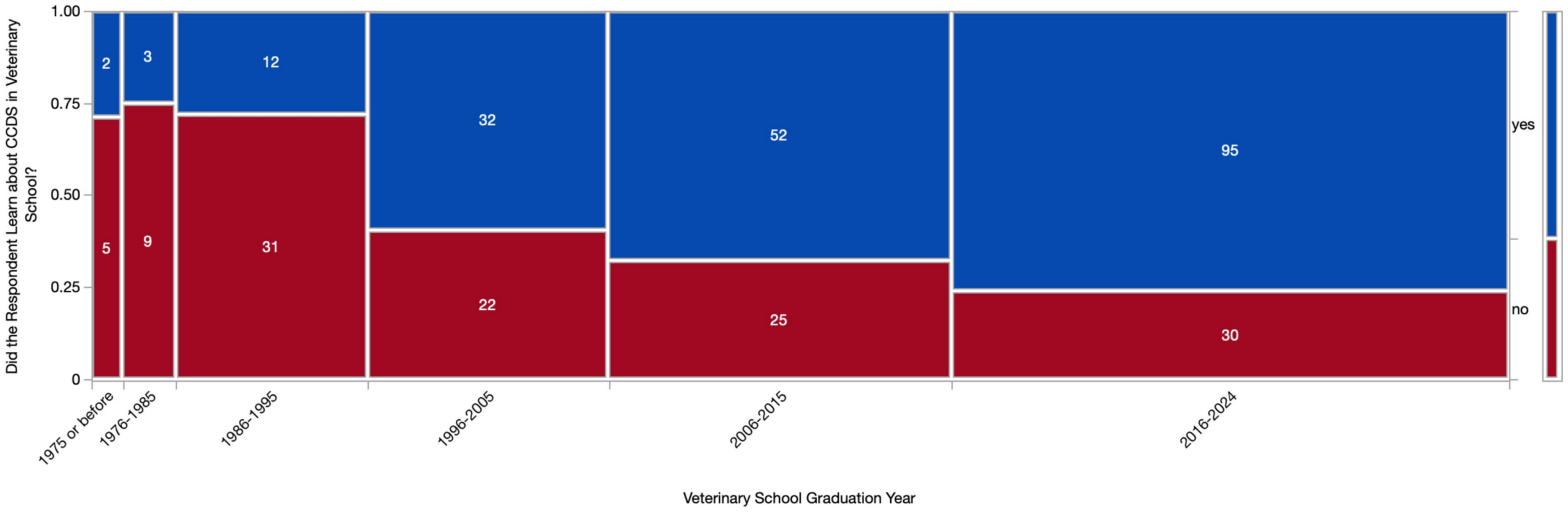
Contingency table of respondents who had and had not learned about Canine Cognitive Dysfunction Syndrome (CCDS) in veterinary school based on graduation year. A Chi square test revealed a significant difference (*p* < 0.0001) in CCDS education based on graduation year.

Most respondents (294/318, 92.5%) learned about CCDS either in veterinary school (196/318, 61.6%) and/or specialized CE training (242/318, 76.1%). There was an association (*p* < 0.0001) between graduation year and whether the respondent had learned about CCDS in veterinary school, with newer graduates being more likely to have learned about CCDS in school. However, the respondents who had not learned about CCDS in school were still represented across all graduation years (Figure 3). Other settings where vets learned about CCDS included: primary (peer-reviewed publications) and secondary (online articles, YouTube videos, social media) literature, mentors/clinical experience, and personal experience with an aging pet.

### Diagnosis of CCDS

**Figure 4:**
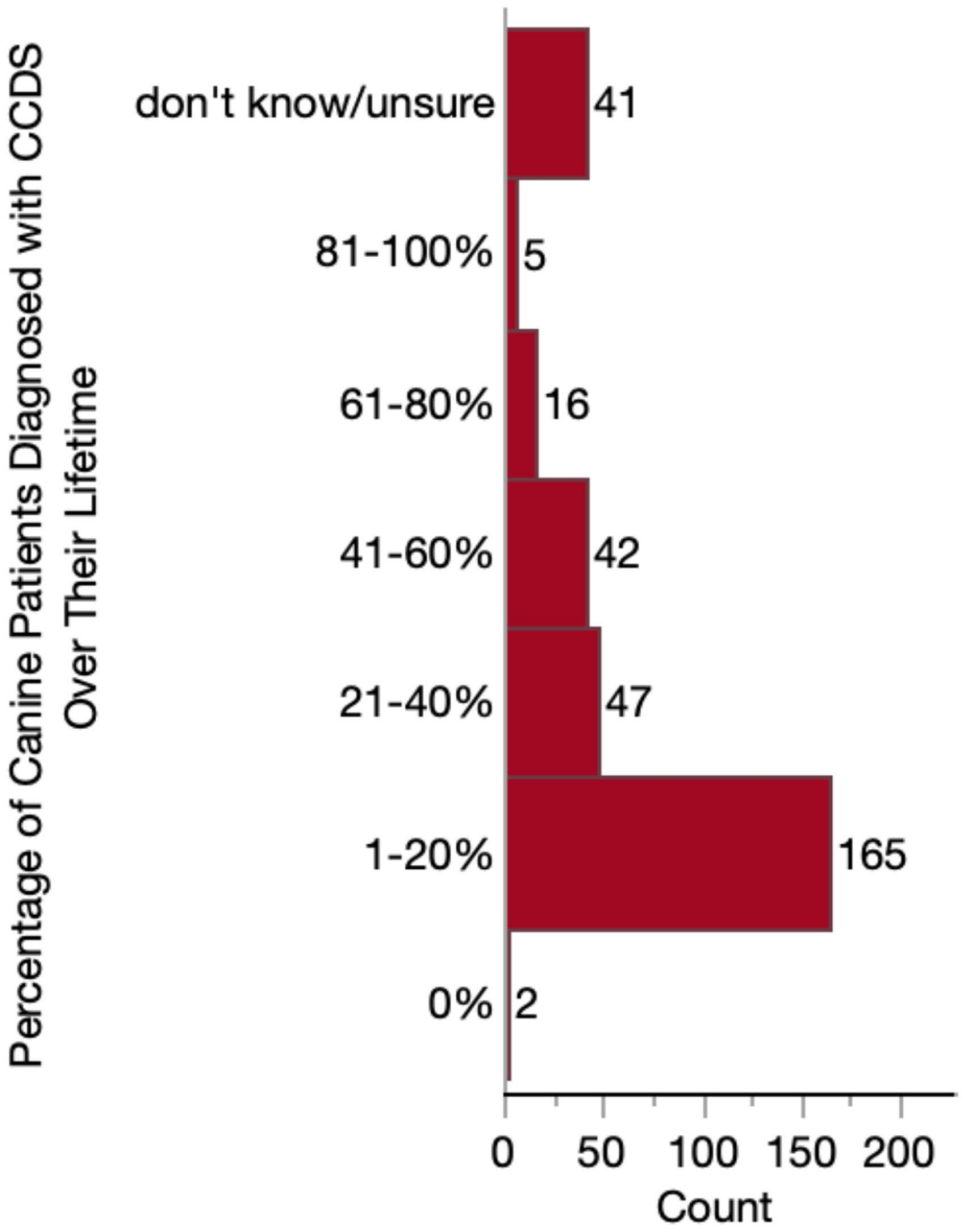
Histogram of the percentage of patients who receive a diagnosis of Canine Cognitive Dysfunction Syndrome (CCDS) at some point in their lifetime.

We first assessed the proportion of respondents’ senior patient population with a CCDS diagnosis, whether made by themselves or by another veterinarian. A little over half of respondents (165/318, 51.9%) reported that only a small percentage (1–20%) of their patients receive a CCDS diagnosis during their lifetime. Only two respondents indicated that none of their patients had ever received a diagnosis. The remaining veterinarians reported higher percentages of diagnosed patients, except for a small subset of respondents (41/318, 12.9%) who reported that they did not know or were unsure. A full summary of these data is detailed in Figure 4.

To identify who specifically was making a CCDS diagnosis, we asked how often veterinarians refer their CCDS patients to a specialist. Most veterinarians reported they rarely (139/318, 43.7%) or never (141/318, 44.3%) referred to a specialist when presented with a CCDS case. To those who indicated that they refer at least occasionally (i.e., selected rarely, sometimes, or often), we asked which type of specialist they referred to. Veterinarians predominantly (131/177, 74.0%) sent their patients to a veterinary neurologist, with fewer referring to a veterinary behaviorist (67/177, 37.9%) or veterinary internist (32/177,18.7%).

**Figure 5:**
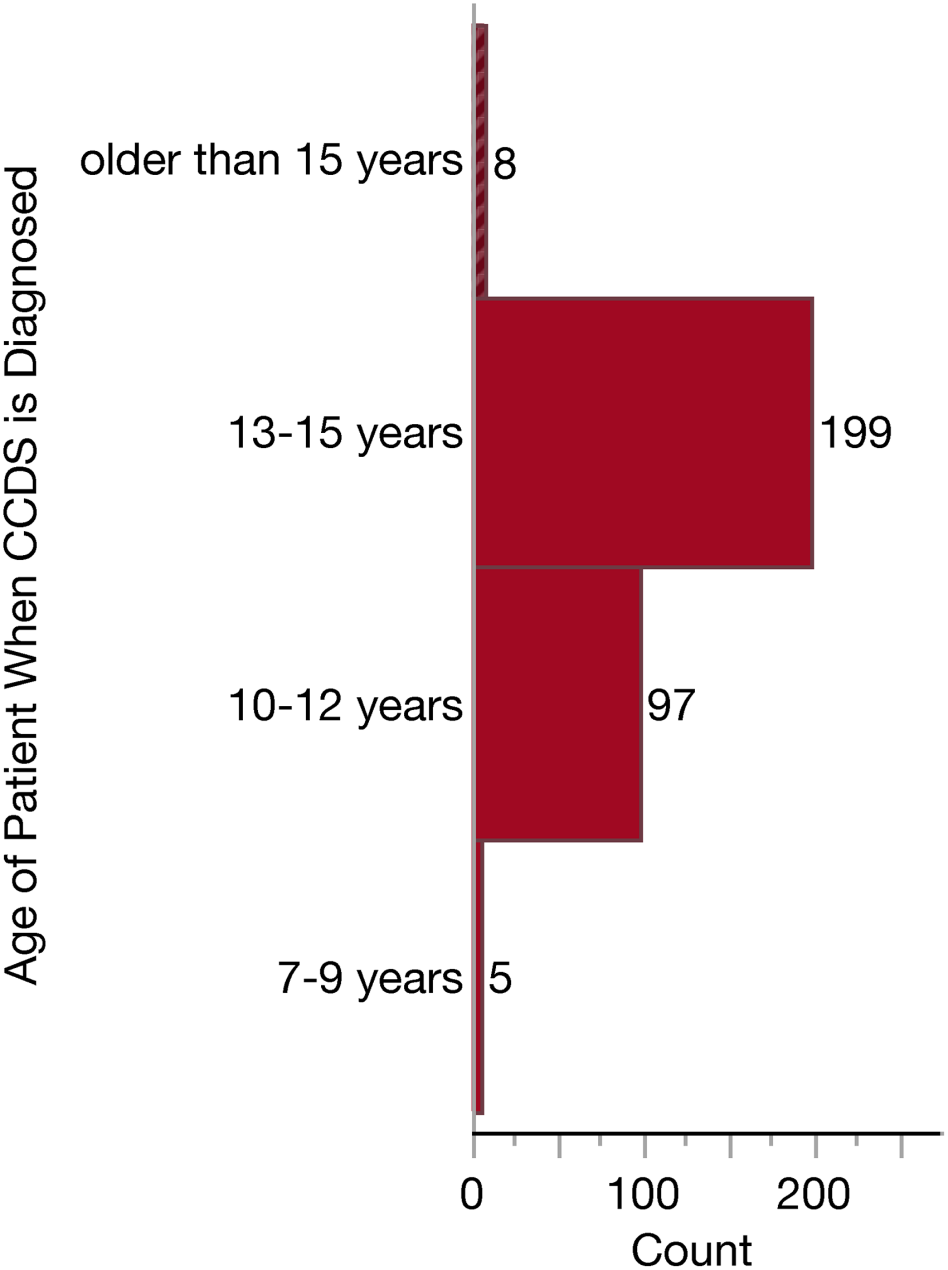
Histogram of the age ranges in which Canine Cognitive Dysfunction Syndrome (CCDS) is diagnosed.

Nearly all respondents (309/318, 97.2%) reported having diagnosed at least one case of CCDS during their career. Therefore, questions surrounding diagnosis frequency, patient age at diagnosis, and diagnostic tools were only presented to these 309 respondents (based on the Qualtrics survey skip logic). To assess the frequency of these diagnoses, we asked these 309 respondents to estimate the number of diagnoses they made annually. The majority reported diagnosing 1–15 dogs per year on average (189/309, 61.2%). Far fewer reported diagnosing between 16 and 25 cases annually (74/309, 23.9%), 26–50 annually (30/309, 9.7%), or more than 50 cases a year (15/309, 4.9%). Only one respondent reported that they make zero diagnoses on average annually. The majority (199/309, 64.4%) of respondents who diagnose CCDS do this in older patients between 13 and 15 years. The second most frequently reported age range for diagnosing a dog with CCDS was 10–12 years (97/309, 31.4%). Substantially fewer veterinarians reported frequently diagnosing CCDS in dogs younger than 10 or older than 15 years of age, as illustrated in Figure 5.

**Figure 6:**
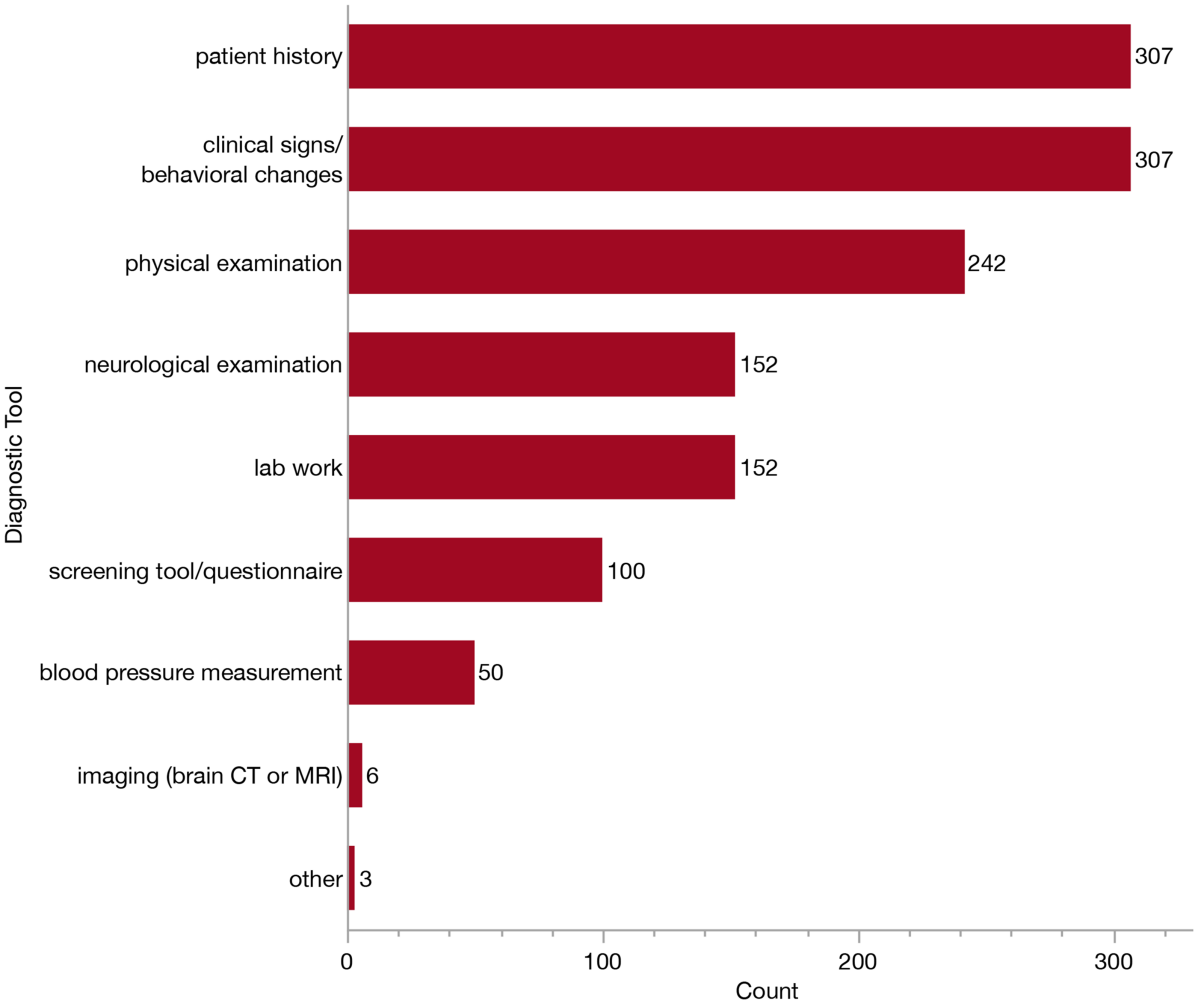
Bar graph of frequency of selection of diagnostic tools when establishing a Canine Cognitive Dysfunction Syndrome (CCDS) diagnosis and/or ruling-out of other differential diagnoses.

To aid in diagnosis, all but one respondent reported using a combination of diagnostic tools. This one veterinarian indicated they relied solely on patient history. The options listed for respondents included diagnostic tools which could support a diagnosis of CCDS and/or rule out other differential diagnoses. Respondents could select as many as they felt appropriate from the following list: patient history, clinical signs/behavioral changes, physical examination, neurological examination, blood pressure measurement, screening questionnaire, serum biochemistry, blood cell count and urinalysis, imaging (brain CT or MRI) and other. The respondents who answered “other” described the following: “rule out other systemic or neurologic disease,” “radiographs to rule out other disease” and “Traditional Chinese Veterinary Medicine (TCVM) work-up.” Because respondents were able to select multiple diagnostic tools, there was a great deal of variability in the combination of tools selected. The most common combination of tools was patient history, clinical signs/behavioral changes and a physical examination (reported by 52/309, 16.8% of respondents). This was followed closely behind by the combination of patient history, clinical signs, physical exam, neurologic exam and lab work (reported by 48/309, 15.5% of respondents). To further understand the relative use of each tool, we examined the independent frequency of selection for each option, as summarized in Figure 6. All but two veterinarians rely upon patient history and clinical signs/behavioral changes to inform their diagnosis, and most veterinarians (242/309, 78.3%) conduct a physical examination. About half of veterinarians (152/309, 49.2%) conduct a neurological examination, and the same number reported conducting lab work in their diagnostic workup, whereas about one-third (100/309, 32/4%) of respondents use a screening tool or questionnaire. Far fewer will obtain a blood pressure measurement (50/309, 16.2%) or advanced imaging (6/309, 1.9%) to help narrow their differential diagnoses.

**Figure 7:**
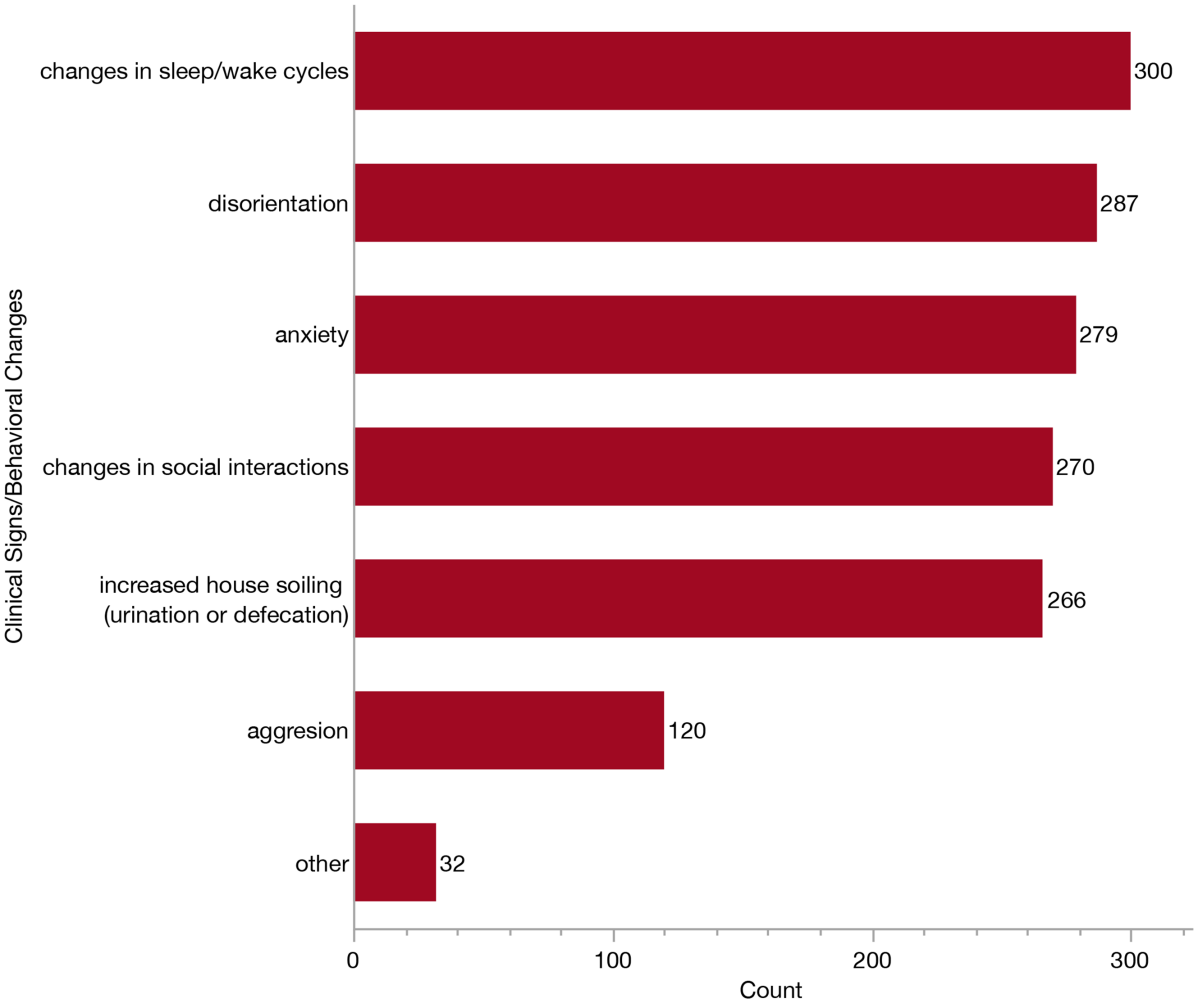
Bar graph of frequency of selection of clinical signs/behavioral changes when establishing a Canine Cognitive Dysfunction Syndrome (CCDS) diagnosis.

Veterinarians who reported relying on clinical signs and behavioral changes to inform their diagnosis (*n* = 307) were presented with a follow-up question where they were asked to indicate which specific signs they consider from the following: changes in sleep/wake cycles, changes in social interaction, disorientation, increased house soiling (urination or defecation), anxiety, aggression or other. Again, respondents could select all that apply. The majority of these veterinarians selected all of the listed clinical signs (97/307, 31.6%), or all signs except for aggression (90/307, 29.3%). Specific frequencies of each of these clinical signs/behavioral changes are provided in Figure 7. The most relied upon behavioral change was disturbances to sleep/wake cycles (reported by 300/307, 97.7%). In contrast, aggression was only selected as a sign by 39.1% (120/307) of veterinarians. The “other” clinical signs described included: pacing/compulsive behaviors, appetite changes, staring, loss of trained behaviors, circling (without a head tilt), getting stuck in corners, changes in vocalization, sensory changes, resistance to restraint and lack of purposeful movement.

**Figure 8:**
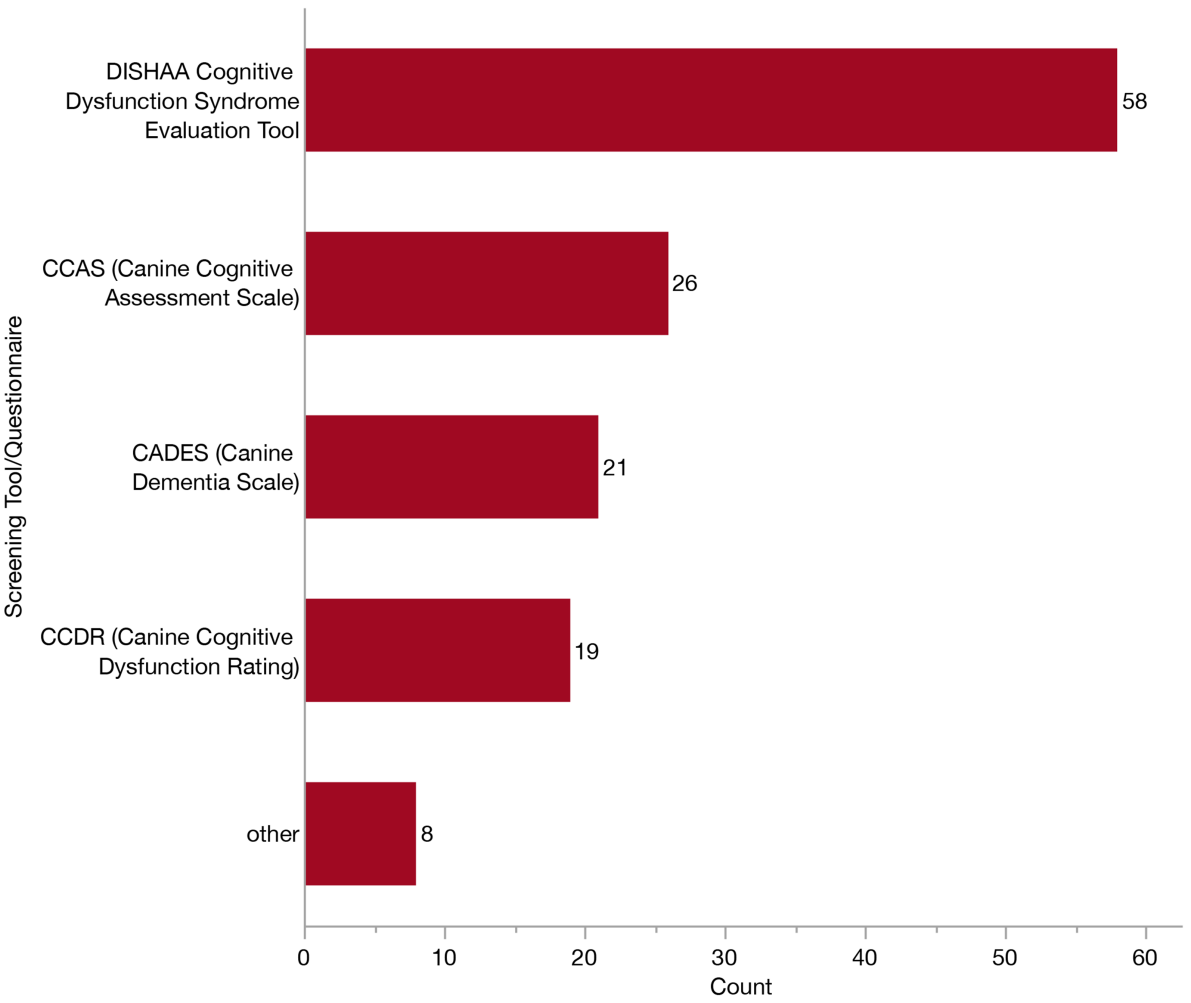
Bar graph of frequency of selection of screening tools/caregiver questionnaires when establishing a Canine Cognitive Dysfunction Syndrome (CCDS) diagnosis.

The 153 veterinarians who indicated using laboratory work in their diagnostic workup (to rule out other differential diagnoses) were presented with a follow-up survey question where they were asked to select which test (s) they employ. Most veterinarians (118/153, 77.1%) reported running a combination of complete blood count (CBC), serum biochemistry (Chem) and urinalysis (UA). Veterinarians who reported relying on a screening tool or questionnaire (*n* = 100) were displayed a subsequent question where they were asked to indicate which specific ones they employ from the following: Purina’s DISHAA Cognitive Dysfunction Syndrome Evaluation Tool, Canine Cognitive Dysfunction Rating (CCDR), Canine Dementia Scale (CADES), Canine Cognitive Assessment Scale (CCAS), and other. Most veterinarians relied exclusively upon Purina’s DISHAA Cognitive Dysfunction Syndrome Evaluation Tool (43/100, 43%), although 28% (28/100) reported using several. Specific frequencies of each of these are provided in Figure 8.

We asked all respondents (*n* = 318) if there was any single thing that would make them more confident in their diagnosis of CCDS. Most respondents (203/318, 63.8%) reported they would like standardized diagnostic criteria or guidance on distinguishing CCDS from other age-related disease. This was followed by far fewer who reported they would like more specialized training or continuing education opportunities (50/318, 15.7%), additional screening tools (20/318, 6.3%), accessible primary resources (13/318, 4.1%), online resources (12/318, 3.8%), formal referral pathways to veterinary specialists (4/318, 1.3%), or other (8/318, 2.5%). Most who selected “other” indicated they would like a combination of the previously mentioned resources, while one individual described wishing there was a specific scoring system which could be employed. Few vets (2.5%, 8/318) selected no option.

### Management of CCDS

To investigate how CCDS is managed/treated in the veterinary clinic, we only included respondents who had encountered a patient with CCDS in their practice (*n* = 316); the survey was concluded for the remaining two respondents. Initially, we sought to understand veterinarians’ overall impression of treatment strategies. Regardless of whether recommended by themselves or another veterinarian (i.e., a specialist), respondents were asked to rate the overall effectiveness of current treatment/management strategies. Most veterinarians felt that current strategies were slightly (179/316, 56.6%) or moderately (112/316, 35.4%) effective. Far fewer felt they were not effective at all (21/309, 6.6%) or very effective (4/316, 1.3%).

**Figure 9:**
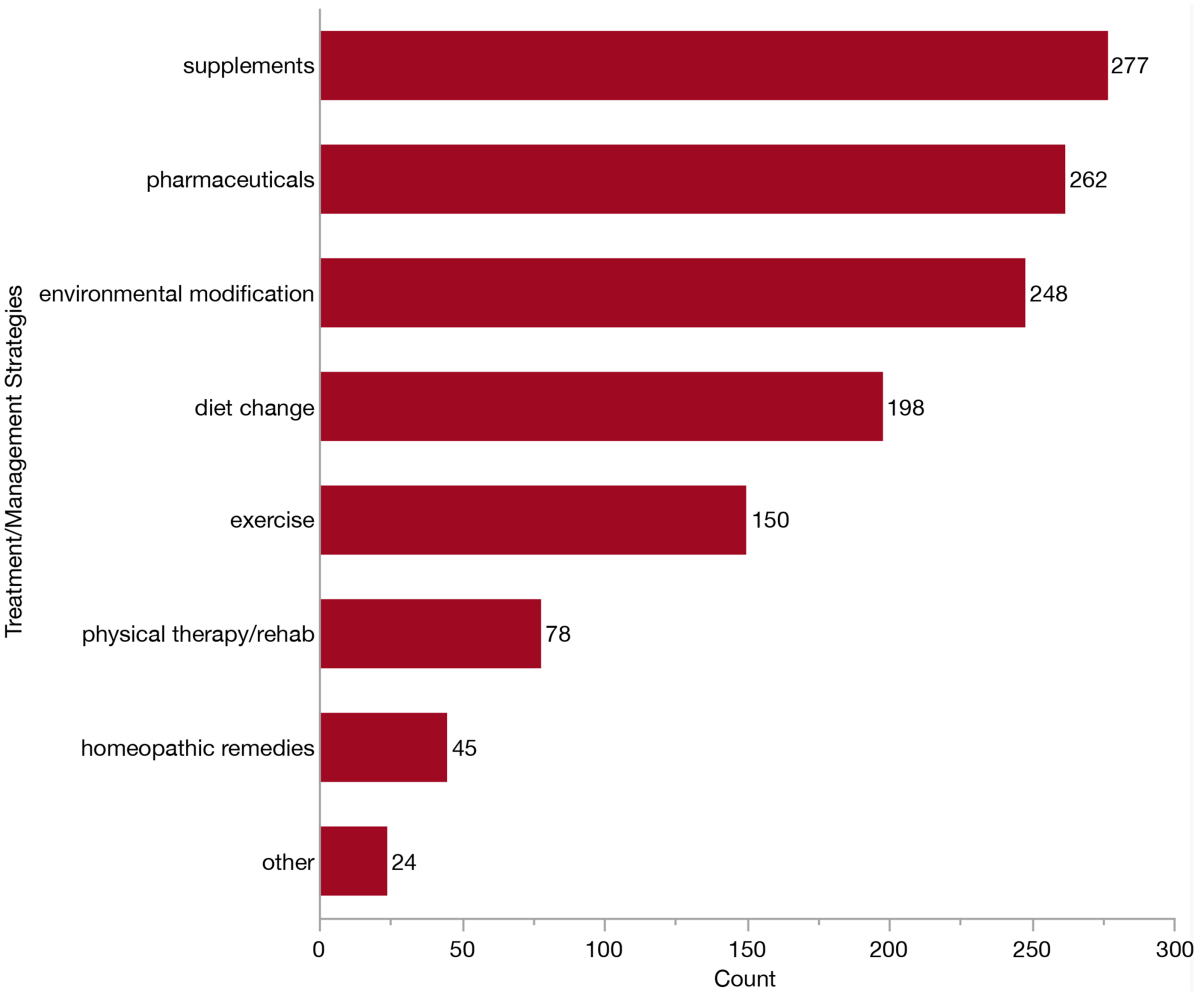
Bar graph of frequency of selection of commonly recommended strategies for treatment/management of Canine Cognitive Dysfunction Syndrome (CCDS).

We next examined specific management strategies, by asking how many of these clinicians were routinely recommending the following: pharmaceuticals, supplements, diet change, environmental modification, exercise, physical therapy, homeopathic remedies or other. At this prompt, an additional three veterinarians reported that they had never managed a dog with CCDS. One veterinarian reported not typically recommending anything upon diagnosis. Consequently, 312 veterinarians were displayed follow-up questions surrounding treatment recommendations/preferences and included in the corresponding descriptive analyses. The majority of these respondents (302/312, 96.8%) reported using a combination of these strategies (though not necessarily in the same patient). The most common combination involved the use of pharmaceuticals, supplements, diet change and environmental modification, reported by 10.3% (32/312). The second most common combination (30/312, 9.6%) included all the same as above, with the addition of exercise. To further understand the relative use of each management strategy, we examined the independent frequency of selection in Figure 9. A slightly greater number of veterinarians recommend supplements (277/312, 88.8%) compared to pharmaceuticals (262/312, 83.9%), although these were the two most indicated management strategies. Environmental modification was the next most frequently reported approach, cited by 79.5% (248/312) of veterinarians. Diet change was indicated by about two-thirds of respondents (198/312, 63.5%), and exercise was selected by a little less than half (151/312, 48.4%). Far fewer selected physical therapy/rehabilitation (78/312, 25.0%), homeopathic remedies (45/312, 14.4%), or other (24/312, 7.7%). Of the respondents who selected “other,” thirteen described using brain games or enrichment (i.e., food puzzles, lick pads, sniffing, snuffle mats and toys). Two veterinarians indicated the use of acupuncture. Additional individual “other” responses included anxiolytics, cannabidiols (CBD), cognitive therapy, owner education, pain control, pet contact, TCVM treatment/herbal medications, and treatment of concurrent diseases (each *n* = 1).

**Figure 10:**
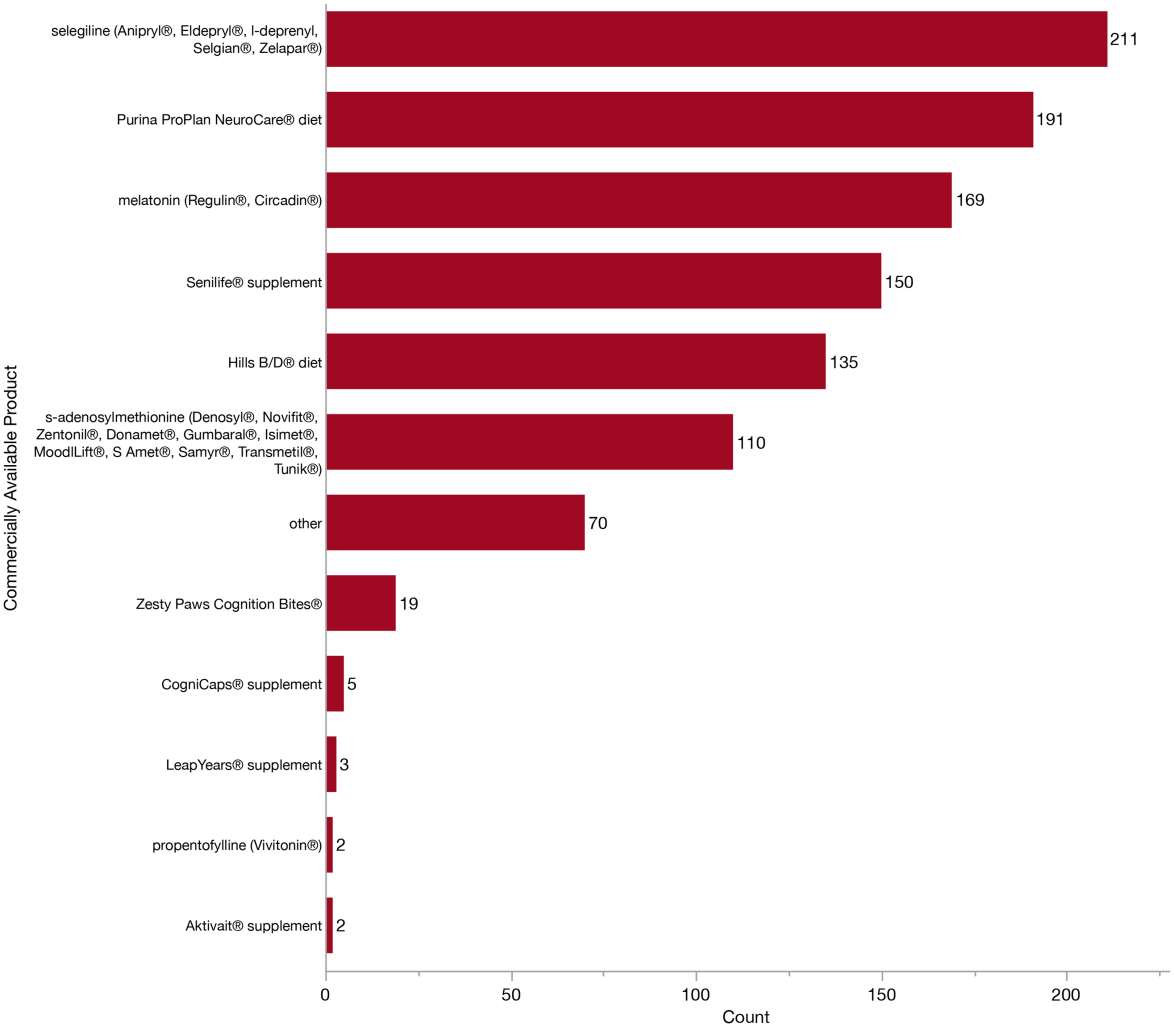
Bar graph of frequency of selection of commercially available products which are marketed towards patients with Canine Cognitive Dysfunction Syndrome (CCDS).

Because there are many commercially available diets, pharmaceuticals, and supplements currently marketed toward aging dogs with CCDS, we next wanted to know which specific products veterinarians were using and finding to be effective. Specifically we asked about the use of Aktivait® supplement, CogniCaps® supplement, Hills B/D diet, LeapYears® supplement, melatonin (Regulin®, Circadin®), propentofylline (Vivitonin®), Purina ProPlan NeuroCare® diet, s-adenosylmethionine (Denosyl®, Novifit®, Zentonil®, Donamet®, Gumbaral®, Isimet®, MoodlLift®, S Amet®, Samyr®, Transmetil®, Tunik®), selegiline (Anipryl®, Eldepryl®, l-deprenyl, Selgian®, Zelapar®), Senilife® supplement, and Zesty Paws Cognition Bites®. Respondents could select as many products as they felt appropriate. All 312 veterinarians participating in the treatment section of the survey received this question, regardless of their previous selection for diet change, pharmaceuticals or supplements, as we anticipated that listing specific products might aid recall. The majority (284/312, 91.0%) of veterinarians selected multiple from the list of commercially available products. However, it is important to note that veterinarians could not specify whether products were used concurrently in the same patient, rather it is more likely that different products are recommended to different patients. Accordingly, we assessed the individual frequency of use of each product in Figure 10. The most frequently indicated product was selegiline (211/312, 67.6%). Many veterinarians (191/312, 61.2%) also indicated using Purina ProPlan NeuroCare® diet and melatonin (169/312, 54.2%). A little less than half of veterinarians recommend the Senilife® supplement (150/312, 48.1%) and Hills b/d® diet (135/312, 43.3%), whereas about a third recommend s-adenosylmethionine (110/312, 35.3%). Far fewer veterinarians indicated Zesty Paws Cognition Bites® (19/312, 6.1%). CogniCaps® supplement (5/312, 1.6%), LeapYears® supplement (3/312, 1.0%), Aktivait® supplement (2/312, 0.6%), or propentofylline (Vivitonin®) (2/312, 0.6%). Nine veterinarians (9/312, 2.9%) left the question blank and did not write in any other responses, therefore implying they do not use any specific product. Whereas 22.4% (70/312) did select “other” and described a broad range of additional diets, pharmaceuticals, and supplements. To preserve the specificity (or lack thereof) of the original responses, we have maintained the wording by veterinarians without alteration. Incidentally, this may reflect a difference between veterinarians, with some considering product recommendations at the class-level, while others may feel more compelled by specific products. The following products were identified: acepromazine (*n* = 1), anxiolytics (*n* = 2), amantadine (*n* = 1), Anxitane® (*n* = 1), CBD (*n* = 3), Cholodin® (*n* = 2), Clomipramine (*n* = 1), Coq10 (*n* = 1), Denamarin® Advanced (*n* = 1), Dr. Buzby’s Brain Boost™ (*n* = 1), EFA supplement (*n* = 3), ElleVet™ (*n* = 2), fish oil (*n* = 2), fluoxetine (*n* = 1), gabapentin (*n* = 11), Hill’s Senior Vitality (*n* = 2), Huperzine A (*n* = 1), keppra (*n* = 2), lion’s mane mushroom (*n* = 2), natural calming agents (*n* = 1), Neutricks (*n* = 1), Nutrix diet (*n* = 1), omega 3 fatty acid supplements (*n* = 4), Omega Benefits® (*n* = 1), ProNeurozone® (*n* = 1), Proquiet® (*n* = 1), Proviable® probiotic (*n* = 1), Purina Bright Minds diet (*n* = 18), Royal Canin® Mature Consult diet (*n* = 1), Solliquin® (*n* = 3), SSRIs (*n* = 1), Stasis in Mansion of Mind herbal by Jing Tang (*n* = 1), trazodone (*n* = 3), TCVM herbals (*n* = 2), TriPlex™ MCT oil (*n* = 1), Ursolyx™ (*n* = 1), VetriScience® Golden Years (*n* = 1), VetriScience® Senior (*n* = 1), Welactin® Advanced (*n* = 1), Zylkene® (*n* = 2).

In order to get insight into which interventions respondents perceive as most effective, veterinarians were asked to identify the single most effective management strategy and the single most effect product for managing CCDS in their patients. There was alignment across most effective management strategy and product (i.e., those who selected diet modification to be most effective also selected a specific diet as the most effective product). The largest number of respondents (135/312, 43.3%) selected pharmaceuticals as the most effective management strategy. Of these, almost half of respondents (67/135, 49.6%) indicated that selegiline was the most effective pharmaceutical. Environmental modification was the next most effective management strategy reported by respondents (54/312, 17.3%). In turn, 40.7% (22/54) of these respondents reported that they either do not use any products or do not find any products to be effective. Supplements were the third most popular choice (50/312, 16.0%), with most of these respondents selecting either Senilife® (16/50, 32.0%) or melatonin (12/50, 24.0%). Veterinarians who selected diet change were the most consistent across their responses, with 79.3% (23/29) of veterinarians also listing a specific diet as most effective (including Purina ProPlan NeuroCare®, Purina ProPlan Bright Minds® and Hills b/d®).

However, there were also some disagreements between these selections. For example, 14.1% (19/135) of veterinarians who selected pharmaceuticals as most effective in CCDS management went on to select a supplement or diet as the single most effective product. Further, 18.5% (25/135) of these individuals stated that they did not use any of the listed products (or find them to be effective), despite selecting pharmaceuticals as the most effective CCDS management strategy. Similarly, 16.0% (8/50) of veterinarians who stated supplements to be most effective in managing CCDS, went on to select selegiline as the most effective product. Regardless of selected management strategy, selegiline was the most frequently identified single most effective product (91/312, 29.2%). This was followed by Senilife® (35/312, 11.2%), melatonin (29/312, 9.3%) and Purina ProPlan NeuroCare® diet (23/312, 7.4%). All other products were endorsed by a notably smaller numbers of respondents. A total of 14.4% (45/312) of respondents indicated that they do not find any of the available products to be effective. However, almost all these respondents (39/45, 86.7%) previously reported that they recommend two or more of these same products. Additionally, fifteen veterinarians (15/312, 4.8%) were unsure and could not indicate a specific product to be most effective.

**Figure 11:**
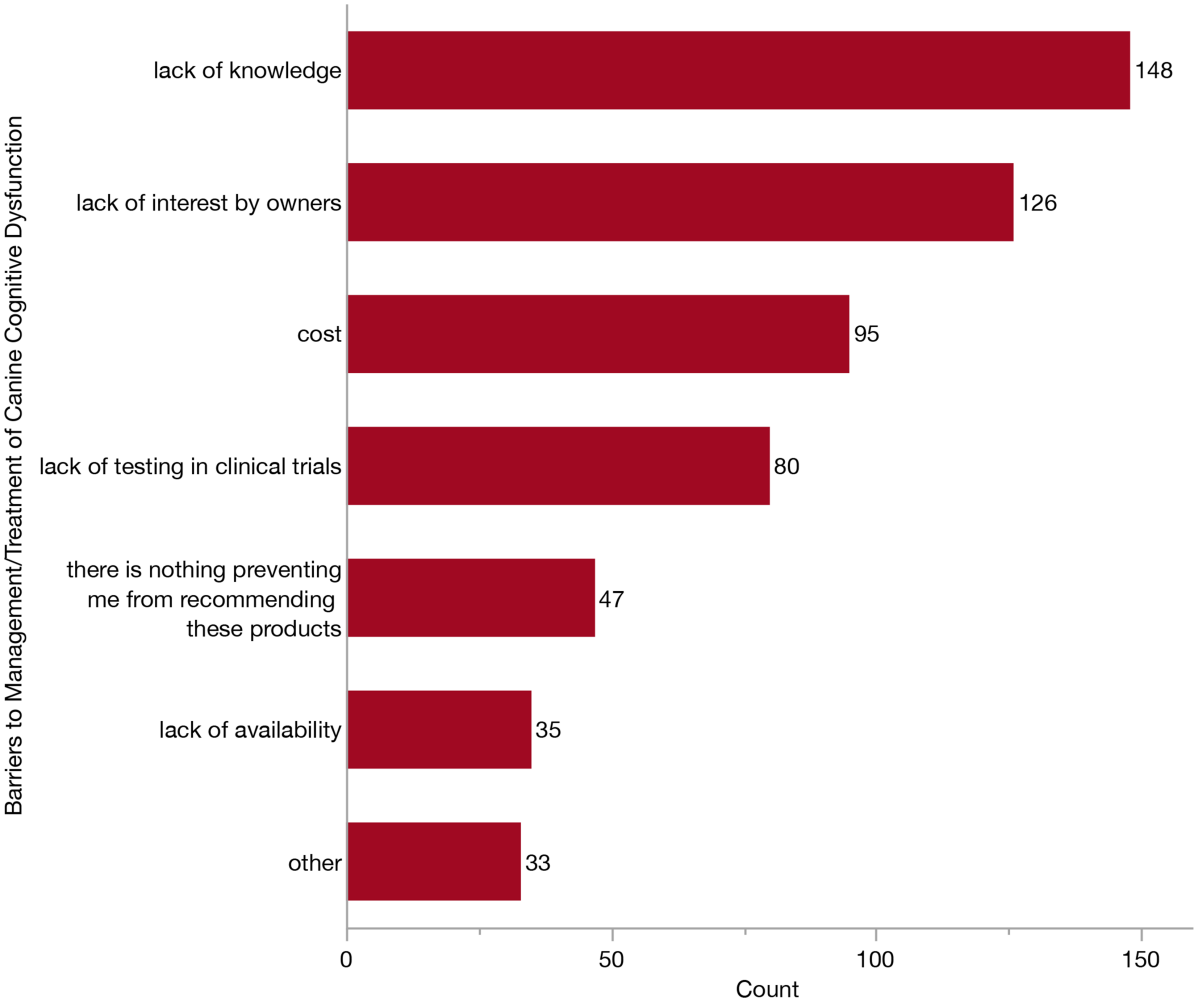
Bar graph of barriers preventing the recommendation of commercially available products marketed toward patients with Canine Cognitive Dysfunction Syndrome (CCDS).

To better understand the hesitancy and barriers preventing veterinarians from recommending currently available products, we asked respondents to select as many barriers as they felt applied from the following: cost, lack of availability, lack of interest from owners, lack of knowledge, lack of testing in clinical trials and other. This question was presented to all veterinarians except the two who had initially reported that zero of the patients in their practice had ever been diagnosed with CCDS (*n* = 316). Notably, the most frequently selected response, even when considering both varying combinations and singular selections, was a lack of knowledge (47/316, 14.9%). To explore this further, we investigated the frequency of each individual response, whether reported individually or in combination with other responses (Figure 11). The top three most frequently reported were lack of knowledge (148/316, 46.8%), lack of interest from owners (126/316, 39.9%), and cost (95/316, 30.1%). This was followed by lack of testing in clinical trials, lack of availability and “other.” Some veterinarians (47/316, 14.9%) stated there was nothing which prevented them recommending these products.

### Veterinarian’s understanding of client perceptions

**Figure 12:**
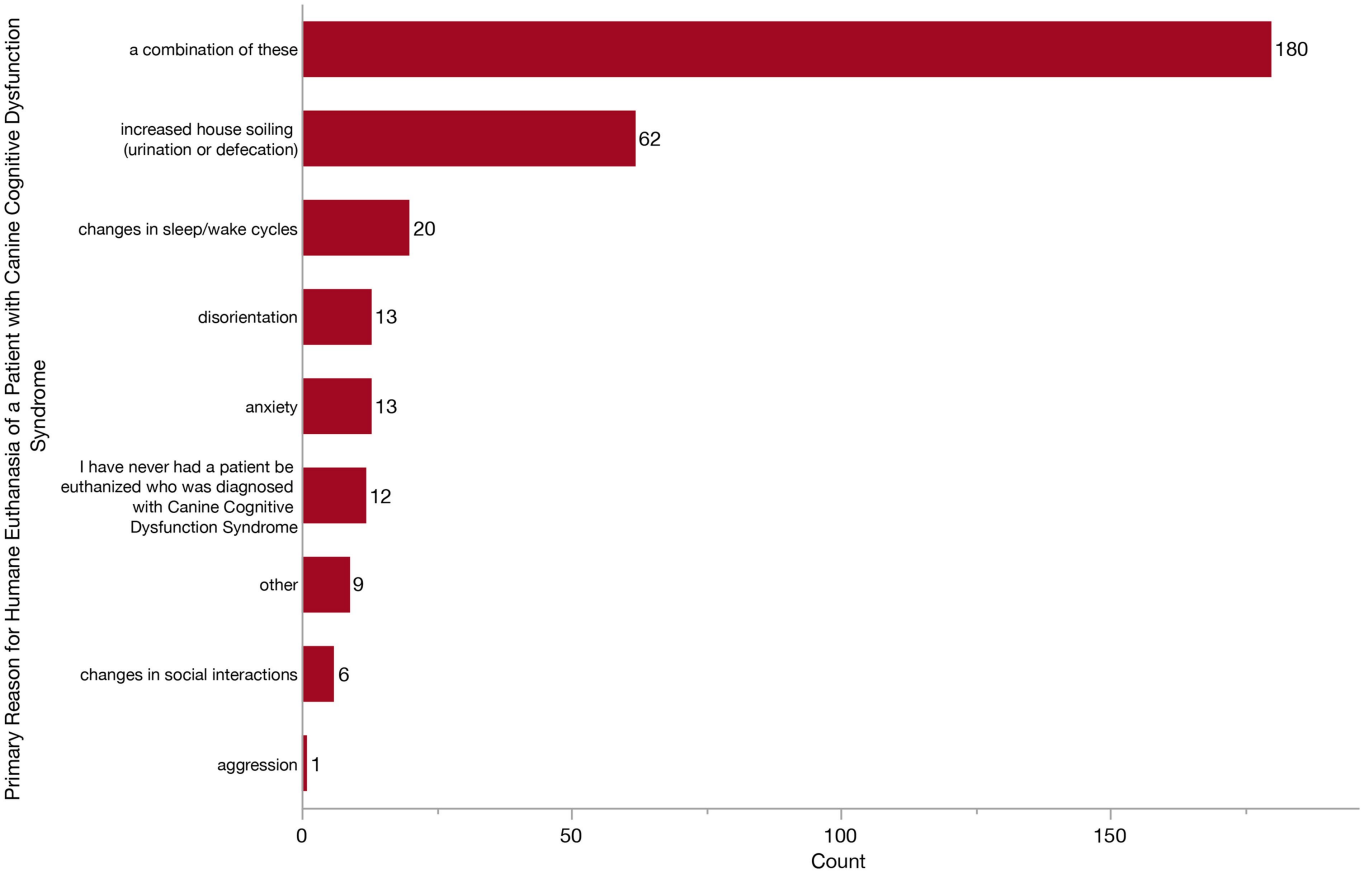
Bar graph of the primary reason for owners to elect euthanasia of a dog with Canine Cognitive Dysfunction Syndrome (CCDS) from the veterinarian’s perspective.

Because clients also play a critical role in the management of their senior pets, we asked all remaining respondents (i.e., those that have encountered a CCDS patient in their practice, even if they have not personally treated/managed them, *n* = 316) to what extent their clients are concerned about CCDS. Respondents reported a differing pattern to themselves, with most selecting that their clients were either only moderately (138/316, 43.7%) or slightly (105/316, 33.2%) concerned about CCDS in their senior dogs. Fewer felt that owners were very concerned (59/316, 18.7%), extremely concerned (7/316, 2.2%) or not concerned at all (7/316, 2.2%). When asked who most frequently initiates a conversation about CCDS, respondents were fairly split between the veterinarian (147/316, 46.5%) and the client (114/316, 38.0%). Fewer respondents reported that it depends (48/316, 15.2%), and only two veterinarians reported that it was the veterinary technician/nurse who initiates the conversation. Regarding the resources utilized to facilitate CCDS discussions, veterinarians most often offer their clients a handout (171/316, 54.1%) or refer them to a website (77/316, 23.4%). Far less frequently, they will offer primary resources (such as a peer-reviewed article) or recommend a CCDS support group. About one third of veterinarians (108/316, 34.2%) do not provide their client with any resources (Figure 12).

Finally, we wanted to see whether veterinarians were observing any noticeable trends across specific CCDS changes which owners feel warrant humane euthanasia. We asked what they feel is the primary reason that owners of dogs with CCDS elect to euthanize. Respondents could answer from the following: disorientation, changes in social interaction, changes in sleep/wake cycles, house soiling (urination or defecation), anxiety, aggression, or a combination of factors. Most veterinarians (180/316, 57.0%) responded that it was a combination of factors (Figure 12). However, of those who responded with a single factor, increased house soiling was the most frequent (62/316, 19.6%) followed by changes in sleep/wake cycles, anxiety, disorientation and changes in social interactions. Twelve veterinarians (3.8%) reported never having a patient with CCDS be euthanized.

## Discussion

In this study, we set out to describe how veterinarians are currently diagnosing and managing CCDS in clinical practice. We obtained 318 responses from veterinary clinicians in the US who see dogs in their routine patient population, the majority of whom were general practitioners. While nearly all respondents had diagnosed CCDS at some point in their careers, the frequency of diagnosis appeared lower than expected, with most veterinarians reporting relatively few (1–15) cases diagnosed per year. These diagnoses are most often happening in dogs aged 13–15 years old. To establish a diagnosis, all but two veterinarians reported relying on patient history and clinical signs or behavioral changes. However, no single clinical sign emerged as the most common indicator. Over 80% of veterinarians reported recommending supplements or pharmaceuticals when managing patients with CCDS. When asked to identify the most effective intervention, the majority cited pharmaceuticals, most commonly selegiline, as the most effective treatment option. These data can serve as a guide to better establish practical consensus as to which diagnostic tools and treatments are most relevant to primary care practice.

We found that most veterinarians (over 90%) reported prior exposure to educational content on CCDS, either during veterinary school or through specialized continuing education training. Despite this, approximately half of respondents still reported a lack of knowledge regarding available treatment options, suggesting a disconnect between education and clinical confidence or application. Expansion of curriculum specific to geriatric medicine and CCDS may enhance the knowledge bases of future veterinarians. Another potential contributor to this gap may be the underutilization of key diagnostic tools. For example, only about half (~49%) of veterinarians reported performing a neurological examination when assessing a CCDS patient and less than 2% perform imaging. Given that CCDS is a diagnosis of exclusion, a neurological exam and/or imaging may be essential to ensure the rule-out of other conditions that could account for similar age-related behavioral changes (30). While imaging is expensive and not always available in all clinical settings, a neurological examination is relatively accessible and easy to perform. However, its limited use may reflect a phenomenon known as neurophobia, which has been documented in both human and veterinary medicine and refers to an aversion toward clinical neurology and the neurosciences among clinicians (40–42). A similar trend has been observed in veterinary behavioral medicine, where many clinicians report feeling underprepared to manage behavioral cases (43). Despite this, the majority of veterinarians in our study appear to be managing CCDS cases themselves, rather than referring to veterinary specialists (neurologists, behaviorists or internists), consistent with a recent study by Haake et al. (44). These findings highlight the prominent role of general practitioners in diagnosing CCDS. Therefore, efforts should be made to improve education and confidence in these skills, especially among general practitioners, to appropriately identify and manage CCDS.

When asked directly if there was anything that would improve their diagnostic confidence, only 2.5% of respondents selected no options, indicating that the vast majority of veterinarians felt as though additional aids could enhance their confidence. Approximately 64% indicated that they would like standardized diagnostic criteria and guidelines for discriminating CCDS from other differentials. In human medicine, there exist several guidelines for AD (across different working groups) which incorporate clinical cognitive changes, biomarker evidence and rule-out of non-dementia differentials (10–12, 17). However, even with these formally defined guidelines, many patients still go undiagnosed (45–47). This highlights the inherent difficulty of diagnosing a syndrome with gradual onset and variable progression in elderly individuals, even in well-resourced settings. Similarly, while CCDS is thought to affect a substantial proportion of senior dogs, it has been reported that a much smaller proportion gets formally diagnosed by a veterinarian (44, 48). It remains unclear whether this underdiagnosis reflects a true failure to identify symptomatic cases, whether some dogs die before cognitive signs become apparent, or whether diagnosis is deprioritized in the context of more pressing comorbidities, caregiver burden, or limited treatment options. These discrepancies may also reflect a disconnect between the veterinarian communicating the diagnosis and an owner understanding or accepting the diagnosis to the extent they are able to report it in a survey. Still, there exist resources to aid in CCDS screening which are appearing to be under-utilized, with only about a third of veterinarians using a screening questionnaire, similar to that reported in a recent Australian survey (49). Until the field establishes specific guidelines, these questionnaires offer a validated framework for linking observed behavioral changes with probable diagnosis of CCDS once systemic conditions have been ruled out (4, 5, 29, 50).

When considering all interventions, about 90% of veterinarians felt that current treatment strategies were only slightly or moderately effective. Over 80% of veterinarians recommend using pharmaceuticals or supplements, with selegiline being the most frequently recommended product. This is consistent with a recent study performed in Australia which determined that CCDS was most commonly managed by medications (selegiline, propentofylline), environmental modifications, and anti-anxiety treatments (49). After selegiline, the most recommended products in our survey were Purina ProPlan NeuroCare® and melatonin. Both selegiline and diets enriched with medium-chain triglycerides have shown some efficacy in supporting cognitive function in senior dogs (36, 51, 52). Conversely, while there has been some evidence that melatonin attenuates cognitive impairments in humans, this has yet to be specifically studied in dogs with CCDS (53). This aligns with ~25% of respondents who indicated that a lack of testing in clinical trials was a barrier to them recommending specific products, underscoring the need for both expanded clinical research and improved dissemination of existing evidence. However, almost half of respondents (~47%) cited lack of knowledge as a barrier to recommendation, consistent with the discordance observed between specific products recommendations and management strategies selected by respondents. For example, some veterinarians, selected pharmaceuticals as most effective overall strategy and then selected a supplement as the single most effective product or vice versa. This may reflect either lack of clarity between which products meet the definition of a supplement versus a pharmaceutical or, more likely, reflect a broader ambiguity as to what is effective (whether based on anecdotal evidence or limited testing in clinical trials). Further, 39 respondents indicated that they do not find any commercially available products to be effective, despite indicating that they recommend at least one of the products listed in the survey, reflective of a discrepancy between products being recommended and those perceived as effective. Broader discordance is additionally supported by the frequency of write-in responses describing use of non-specific therapies, generalized uncertainty, and/or emphasizing patient/owner specific factors which dictate selection of treatment and its impact. All together, these inconsistencies underscore the uncertainty and varying opinions surrounding efficacy of these products.

While we did not specifically list any anxiolytics in our questions surrounding the treatment of CCDS, many respondents used the “other” selection to identify their use. Given the established connection between dementia and anxiety across both humans and dogs (54–56), future studies could investigate the utility of anti-anxiety medication in the treatment of CCDS. However, a few veterinarians expressed caution with polypharmacy, citing concern for drug interactions, particularly between selegiline and fluoxetine. In humans, co-administration of an SSRI (selective serotonin reuptake inhibitors, ex: fluoxetine) and an MAO-B (monoamine oxidase B inhibitor, ex: selegiline) have been reported, though rarely, to be associated with cases of serotonin syndrome (57–59). This potential risk has been extrapolated to veterinary patients, although direct evidence of this interaction in dogs remains limited. The risks of polypharmacy in elderly patient populations are also well-documented in human medicine but require further exploration in elderly veterinary patients (60–62).

Another incidental finding from our survey was that the majority of respondents’ practices (~68%) do not have specified senior visits, despite large proportions of patient populations being senior (majority between 21 and 60%). Therefore, clients and veterinarians may not have the opportunity to proactively discuss CCDS and other age-related conditions in an efficient and comprehensive way. Further, there is a disparity between veterinarians, who reported strong to moderate concern for CCDS in their senior dog patients, as opposed to their perception of the level of concern held by clients (only moderate to slight). Effective client communication is essential to bridge this gap, however, based on our survey, these conversations are not always led by the veterinarian, and often wait until specific symptoms are mentioned or until the client initiates the conversation. Given that the single most significant risk factor for CCDS is age (48, 63), providing designated visits for all patients above a certain age, which incorporate CCDS screening, may help both the veterinarian and the client to address these concerns before they become unmanageable.

Our study has some limitations. While our findings were consistent with the recent Australian survey, we ultimately did screen for respondents who were practicing veterinarians in the US which may limit extrapolation to other populations. Another limitation is that given this was a voluntary online survey without incentive, it is highly susceptible to response bias. Likely, practitioners who are especially motivated toward senior patients or interested in CCDS were more motivated to respond to the survey. This is further supported by most respondents (approximately 47%) considering themselves to have a special interest in geriatric medicine. Further, individuals with greater technological proficiency may have been more likely to encounter and complete the survey. This is supported by majority of respondents having graduated in the past decade making them presumably younger and more comfortable with digital platforms. However, the COVID-19 pandemic did rapidly increase technological competence across more demographics, especially those who are considered health professionals (64–66).

While we sought to collect responses from all over the country (based on distribution by several state-related Veterinary Medical Associations) (reported in Supplementary Data Sheet 2), the anonymity of the survey precludes our ability to confirm geographic diversity. Future studies including specific analyses of geographic differences (i.e., by state or urban vs. rural) in diagnostic and management practices could provide insight into factors contributing to practice variation. Moreover, increased participation would strengthen the study as we were unable to reach our goal of 385 responses. Therefore, this study is likely underpowered. Additionally, the sample size estimated in the power analysis was based on a conservative estimate of all veterinarians in the US, rather than specifically only those who routinely see dogs, which may affect the precision of the power analysis. Nevertheless, the diversified distribution and targeted screening strategy support the representativeness of the collected sample for the population of interest. Finally, in this study we focused on the perspectives of veterinarians, but evidence demonstrates that veterinarians and dog owners disagree in their opinions of veterinary care for elderly patients (67). Future studies should examine owner perspectives on the identification and management of CCDS in their pets.

Veterinarians play a key role in identifying age-related disease in companion animals (68–70). In this study, we found that while most veterinarians have diagnosed a dog with CCDS at some point in their career, not many diagnose these cases regularly. To make a diagnosis, veterinarians are using a combination of tools, but most commonly rely on patient history and clinical signs. To treat CCDS, most veterinarians use pharmaceuticals and supplements, and many find selegiline to be the most effective treatment option. However, current treatment strategies were collectively described as only slightly or moderately effective at best. When asked what is preventing their recommendation of currently available treatments, respondents reported a lack of knowledge with almost all respondents desiring more information on CCDS. Together, this highlights the need for increased education and consensus surrounding the diagnosis and management of CCDS. Veterinarians would benefit from clear guidelines which are based on scientific evidence. Efforts should aim to increase veterinarian’s knowledge and familiarization of CCDS in order to fully address any needs and concerns of both patients their owners.

## Data Availability

The original contributions presented in the study are included in the article/Supplementary material, further inquiries can be directed to the corresponding author.

## Footnotes

^1^ https://www.qualtrics.com/blog/calculating-sample-size/
