# Supplementary material for: Current practices for diagnosis and management of Canine Cognitive Dysfunction Syndrome in the United States
Source: Front Vet Sci. 2025 Oct 29;12:1685430. doi: 10.3389/fvets.2025.1685430 (PMC12605212; doi:10.3389/fvets.2025.1685430)
Supplement: Supplementary file 1 [file Data_Sheet_1.docx]

Canine Cognitive Dysfunction Survey

Start of Block: Default Question Block

Informed Consent **North Carolina State University College of Veterinary Medicine Informed Consent** We ask that you complete this questionnaire to allow us insight on how different clinicians manage cases of Canine Cognitive Dysfunction Syndrome (CCDS). All information will be kept strictly confidential and will only be reported as anonymous data unassociated with names. We thank you for your participation in our study to expand our understanding of different diagnostic and management strategies for dogs with CCDS. Please review and agree to the following informed consent before proceeding:   Project: Diagnosis and Management of Canine Cognitive Dysfunction, a pilot study to collect descriptive data. IRB number: 27687 Principal Investigator: Natasha Olby, Vet MB, PhD, Dip ACVIM (Neurology) Professor of Neurology/Neurosurgery at North Carolina State University College of Veterinary Medicine. Purpose of Research: The purpose of this research is establishing an understanding of how Canine Cognitive Dysfunction is currently diagnosed and managed by veterinarians. Duration of Participation: If you decide to take part in this survey, it will take about 10-15 minutes to complete. Description of Procedures Examinations: Complete an anonymous online survey (Qualtrics) regarding diagnosis and management of Canine Cognitive Dysfunction in your practice. Study Benefits: Your participation will help us understand how Canine Cognitive Dysfunction is being diagnosed and managed by veterinarians. Your input will provide valuable insights that can shape future clinical trials, enhance continuing education for veterinarians, and improve specialized training programs focused on canine cognitive health. Potential Risks: There are minimal risks to taking part in this study. Right to Withdraw Participation: This study is voluntary and you have the right to withdraw at any time. Confidentiality: This survey is anonymous and it will not be possible to identify individuals participating. The results of this survey will be presented at scientific meetings and published but all data will be reported as collectives with no identification of individuals. Financial Obligations: There are no financial obligations involved in this survey. Study contacts: Dr. Olby (Principal Investigator) is responsible for coordinating this study and if you have any questions she can be contacted by telephone at 919 513 8286 or email at njolby@ncsu.edu. North Carolina State University Institutional Review Board (IRB): If you have questions about your rights as a participant or are concerned with your treatment throughout the research process, please contact the NC State University IRB Director at IRB-Director@ncsu.edu, 919-515-8754.   By selecting to continue below, you agree that you have read and understand all of the information provided above. You additionally agree to participate in the survey and answer questions honestly and to the best of your abilities.

End of Block: Default Question Block

Start of Block: Practice Information

Q1 In which country do you currently practice?

- United States (1)
- other (2) __________________________________________________

Skip To: End of Survey If In which country do you currently practice? = other

Q2 Are you a veterinarian?

- yes (1)
- no (2)

Skip To: End of Survey If Are you a veterinarian? = no

Q3 Are you currently practicing veterinary medicine?

- yes (2)
- no (3)

Skip To: End of Survey If Are you currently practicing veterinary medicine? = no

Q4 In what year did you graduate veterinary school?

- 2016-2024 (1)
- 2006-2015 (2)
- 1996-2005 (3)
- 1986-1995 (4)
- 1976-1985 (5)
- 1975 or before (6)

Q5 Did you learn about Canine Cognitive Dysfunction Syndrome in veterinary school?

- yes (1)
- no (2)

Q6 Have you learned about Canine Cognitive Dysfunction Syndrome while attending CE or other specialized training?

- yes (1)
- no (2)

Q7 Is there somewhere else that you have learned about Canine Cognitive Dysfunction Syndrome which hasn't already been mentioned?

- yes (please explain) (1) __________________________________________________
- no (2)

Q8 What best describes your current position?

- general practitioner/primary care (1)
- veterinary specialist (AVMA recognized) (2)
- veterinary resident (3)
- veterinary intern (5)
- other (please explain) (4) __________________________________________________

Display this question:

If What best describes your current position? = veterinary resident

Or What best describes your current position? = veterinary specialist (AVMA recognized)

Q9 Which AVMA-recognized specialty or specialty organization are you currently boarded in or training to get boarded in? (click all that apply)

-  American Board of Veterinary Practitioners (1)
-  American Board of Veterinary Toxicology (4)
-  American College of Animal Welfare (5)
-  American College of Laboratory Animal Medicine (6)
-  American College of Poultry Veterinarians (7)
-  American College of Theriogenologists (8)
-  American College of Veterinary Anesthesia and Analgesia (9)
-  American College of Veterinary Behaviorists (10)
-  American College of Veterinary Clinical Pharmacology (11)
-  American College of Veterinary Dermatology (12)
-  American College of Veterinary Internal Medicine (13)
-  American College of Veterinary Microbiologists (14)
-  American College of Veterinary Nephrology and Urology (15)
-  American College of Veterinary Ophthalmologists (16)
-  American College of Veterinary Pathologists (17)
-  American College of Veterinary Preventive Medicine (18)
-  American College of Veterinary Radiology (19)
-  American College of Veterinary Sports Medicine and Rehabilitation (20)
-  American College of Veterinary Surgeons (21)
-  American College of Zoological Medicine (22)
-  American College of Veterinary Emergency and Critical Care (23)
-  American Veterinary Dental College (24)

Display this question:

If Which AVMA-recognized specialty or specialty organization are you currently boarded in or trainin... =  American Board of Veterinary Practitioners

Q9A Choose which best applies:

- shelter medicine (1)
- reptile and amphibian (4)
- exotic companion mammal (5)
- canine and feline (6)
- equine (7)
- fish (8)
- food animal (9)
- dairy (10)
- swine health management (11)
- avian (12)
- beef cattle (13)
- feline (14)

Display this question:

If Which AVMA-recognized specialty or specialty organization are you currently boarded in or trainin... =  American College of Veterinary Internal Medicine

Q9B Choose which best applies:

- cardiology (1)
- small animal internal medicine (4)
- large animal internal medicine (5)
- neurology (6)
- oncology (7)
- nutrition (8)

Display this question:

If Which AVMA-recognized specialty or specialty organization are you currently boarded in or trainin... =  American College of Veterinary Microbiologists

Q9C Choose which best applies:

- virology (1)
- immunology (4)
- bacteriology/mycology (5)
- parasitology (6)

Display this question:

If Which AVMA-recognized specialty or specialty organization are you currently boarded in or trainin... =  American College of Veterinary Pathologists

Q9D Choose which best applies:

- anatomic pathology (1)
- clinical pathology (4)

Display this question:

If Which AVMA-recognized specialty or specialty organization are you currently boarded in or trainin... =  American College of Veterinary Radiology

Q9E Choose which best applies:

- diagnostic imaging (1)
- equine diagnostic imaging (4)
- radiation oncology (5)

Display this question:

If Which AVMA-recognized specialty or specialty organization are you currently boarded in or trainin... =  American College of Veterinary Sports Medicine and Rehabilitation

Q9F Choose which best applies:

- canine (1)
- equine (4)

Display this question:

If Which AVMA-recognized specialty or specialty organization are you currently boarded in or trainin... =  American College of Veterinary Surgeons

Q9G Choose which best applies:

- small animal surgery (1)
- large animal surgery (4)

Q10 At what type of practice do you routinely see patients? (click all that apply)

- general practice (1)
- specialty practice (2)
- academic setting (3)
- in-home/mobile practice (4)
- other (please explain) (5) __________________________________________________

Q11 Do you consider yourself to have a special interest in geriatric medicine?

- yes (1)
- no (2)
- neutral (3)

Q12 Does your practice provide specialized senior visits for elderly pets at your practice?

- yes (1)
- no (2)

Q13 Does your routine patient population include dogs?

- yes (1)
- no (2)

Skip To: End of Survey If Does your routine patient population include dogs? = no

Q14 What percentage of your routine canine population is senior (as defined by your practice)?

- 0-20% (1)
- 21-40% (2)
- 41-60% (3)
- 61-80% (4)
- 81-100% (5)

End of Block: Practice Information

Start of Block: Diagnosing Canine Cognitive Dysfunction Syndrome

Q15 To what extent are you concerned about Canine Cognitive Dysfunction Syndrome in your senior patients?

- not concerned at all (1)
- slightly concerned (2)
- moderately concerned (3)
- very concerned (4)
- extremely concerned (5)

Q16 Have you ever diagnosed a dog with Canine Cognitive Dysfunction Syndrome?

- yes (1)
- no (2)

Skip To: Q20 If Have you ever diagnosed a dog with Canine Cognitive Dysfunction Syndrome? = no

Q17 At what age do you most commonly diagnose dogs with Canine Cognitive Dysfunction Syndrome (regardless of breed)?

- younger than 7 years (1)
- 7-9 years (2)
- 10-12 years (3)
- 13-15 years (4)
- older than 15 years (5)

Q18 Approximately how many cases of Canine Cognitive Dysfunction Syndrome do you diagnose annually?

- 0 (1)
- 1-15 (2)
- 16-25 (3)
- 26-50 (4)
- 51-100 (5)
- 100+ (6)

Q19 Which of the following diagnostic tools have you used to diagnose Canine Cognitive Dysfunction Syndrome? (click all that apply)

- patient history (1)
- clinical signs/behavioral changes (2)
- physical examination (9)
- validated screening tool/questionnaire (3)
- neurological examination (10)
- blood pressure measurement (8)
- lab work (6)
- CSF (cerebrospinal fluid) tap (16)
- imaging (brain CT or MRI) (4)
- other (please explain) (5) __________________________________________________

Display this question:

If Which of the following diagnostic tools have you used to diagnose Canine Cognitive Dysfunction Sy... = validated screening tool/questionnaire

Q19A Which of the following validated screening tools/questionnaires have you used? (click all that apply)

- CADES (Canine Dementia Scale) (1)
- CCDR (Canine Cognitive Dysfunction Rating) (2)
- CCAS (Canine Cognitive Assessment Scale) (3)
- DISHAA Cognitive Dysfunction Syndrome Evaluation Tool (4)
- other (please explain) (5) __________________________________________________

Display this question:

If Which of the following diagnostic tools have you used to diagnose Canine Cognitive Dysfunction Sy... = clinical signs/behavioral changes

And Which of the following diagnostic tools have you used to diagnose Canine Cognitive Dysfunction Sy... = patient history

Q19B If relying on patient history or clinical signs, what are the key indicators or criteria you use for diagnosis? (click all that apply)

- disorientation (1)
- changes in social interactions (2)
- changes in sleep/wake cycles (3)
- increased house soiling (urination or defecation) (4)
- anxiety (5)
- aggresion (6)
- other (please explain) (7) __________________________________________________

Display this question:

If Which of the following diagnostic tools have you used to diagnose Canine Cognitive Dysfunction Sy... = lab work

Q19C Which of the following laboratory tests have you used? (click all that apply)

- complete blood count (1)
- chemistry (4)
- urinalysis (5)
- other (please explain) (6) __________________________________________________

Q20 Is there anything that would make you more confident in your ability to diagnose Canine Cognitive Dysfunction Syndrome?

- specialized training/continued education (CE, workshops, webinars, lectures) (1)
- standardized diagnostic criteria (guidance on distinguishing Canine Cognitive Dysfunction Syndrome from other age-related diseases) (2)
- additional screening tools (3)
- formal referral pathways to veterinary specialists (4)
- accessible primary resources (scientific articles) (7)
- online resources (non-peer reviewed articles, message boards, etc.) (8)
- other (please explain) (9) __________________________________________________
- none of the above (10)

Q21 How often do you recommend a specialist referral for diagnosis or management of Canine Cognitive Dysfunction Syndrome?

- always (1)
- sometimes (2)
- rarely (3)
- never (4)

Display this question:

If How often do you recommend a specialist referral for diagnosis or management of Canine Cognitive... = always

Or How often do you recommend a specialist referral for diagnosis or management of Canine Cognitive... = sometimes

Or How often do you recommend a specialist referral for diagnosis or management of Canine Cognitive... = rarely

Q21A To which type of specialist do you refer Canine Cognitive Dysfunction Syndrome cases? (click all that apply)

- board-certified veterinary neurologist (1)
- board-certified veterinary internist (2)
- board certified veterinary behaviorist (3)
- other (please explain) (4) __________________________________________________

Q22 What proportion of your senior dog patient population eventually gets diagnosed with Canine Cognitive Dysfunction Syndrome at some point in their lifespan (by yourself, another veterinarian at your clinic, or a specialist)?

- 0% - none of the patients in my practice have ever been diagnosed with Canine Cognitive Dysfunction Syndrome (1)
- 1-20% (2)
- 21-40% (3)
- 41-60% (4)
- 61-80% (5)
- 81-100% (6)
- don't know/unsure (7)

Skip To: End of Survey If What proportion of your senior dog patient population eventually gets diagnosed with Canine Cogni... = 0% - none of the patients in my practice have ever been diagnosed with Canine Cognitive Dysfunction Syndrome

End of Block: Diagnosing Canine Cognitive Dysfunction Syndrome

Start of Block: Treatment of Canine Cognitive Dysfunction Syndrome

Q23 How would you rate the effectiveness of the current treatment/management strategies (regardless of recommendation by yourself, another veterinarian or veterinary specialist) for Canine Cognitive Dysfunction Syndrome in your patients?

- not effective at all (1)
- slightly effective (2)
- moderately effective (3)
- very effective (4)
- extremely effective (5)

Q24 When managing Canine Cognitive Dysfunction Syndrome in your patients, do you routinely recommend any of the following? (click all that apply)

- pharmaceuticals (1)
- supplements (2)
- diet change (3)
- exercise (4)
- physical therapy/rehab (5)
- homeopathic remedies (6)
- environmental modification (7)
- other (please explain) (8) __________________________________________________
- I do not typically recommend anything upon diagnosis (9)
- I have never managed a dog with Canine Cognitive Dysfunction Syndrome (10)

Skip To: Q28 If When managing Canine Cognitive Dysfunction Syndrome in your patients, do you routinely recommend... = I do not typically recommend anything upon diagnosis

Skip To: Q28 If When managing Canine Cognitive Dysfunction Syndrome in your patients, do you routinely recommend... = I have never managed a dog with Canine Cognitive Dysfunction Syndrome

Q25 Do you routinely recommend any of the following commercially available products aimed to treat/manage Canine Cognitive Dysfunction Syndrome?

- Selegiline (Anipryl®, Eldepryl®, l-deprenyl, Selgian®, Zelapar®) (1)
- S-adenosylmethionine (Denosyl®, Novifit®, Zentonil®, Donamet®, Gumbaral®, Isimet®, MoodlLift®, S Amet®, Samyr®, Transmetil®, Tunik®) (2)
- Propentofylline (Vivitonin®) (13)
- Melatonin (Regulin®, Circadin®) (3)
- Senilife® Supplement (4)
- CogniCaps Supplement (5)
- Aktivait® Supplement (6)
- LeapYears Supplement (7)
- Zesty Paws Cognition Bites (8)
- Hills B/D Diet (9)
- Purina ProPlan NeuroCare Diet (10)
- other (please explain) (11) __________________________________________________
- none of the above (14)

Q26 When managing Canine Cognitive Dysfunction Syndrome in your patients which of the following do you find to be MOST effective? (select only one)

- pharmaceuticals (1)
- supplements (4)
- diet change (5)
- exercise (6)
- physical therapy/rehab (7)
- homeopathic remedies (8)
- environmental modification (9)
- other (please explain) (10) __________________________________________________

Q27 Which of the following commercially available products aimed to treat/manage Canine Cognitive Dysfunction Syndrome do you find to be MOST effective? (select only one)

- Selegiline (Anipryl®, Eldepryl®, l-deprenyl, Selgian®, Zelapar®) (1)
- S-adenosylmethionine (Denosyl®, Novifit®, Zentonil®, Donamet®, Gumbaral®, Isimet®, MoodlLift®, S Amet®, Samyr®, Transmetil®, Tunik®) (4)
- Propentofylline (Vivitonin®) (5)
- Melatonin (Regulin®, Circadin®) (6)
- Senilife® supplement (7)
- CogniCaps supplement (8)
- Aktivait® supplement (9)
- LeapYears supplement (10)
- Zesty Paws cognition bites (11)
- Hills B/D diet (12)
- Purina ProPlan NeuroCare diet (13)
- other (please explain) (14) __________________________________________________
- I do not use any of the above commercially available products to manage Canine Cognitive Dysfunction Syndrome (15)
- I do not find any of the above commercially available products to be effective (16)

Q28 What (if anything) prevents you from recommending the currently available products aimed to treat/manage Canine Cognitive Dysfunction Syndrome? (click all that apply)

- lack of availability (1)
- lack of testing in clinical trials (2)
- lack of knowledge (3)
- lack of interest by owners (4)
- cost (7)
- other (please explain) (5) __________________________________________________
-  there is nothing preventing me from recommending these products (6)

End of Block: Treatment of Canine Cognitive Dysfunction Syndrome

Start of Block: Senior Pet Clients

Q29 To what extent are your clients concerned about Canine Cognitive Dysfunction Syndrome in their senior pets?

- not concerned at all (1)
- slightly concerned (2)
- moderately concerned (3)
- very concerned (4)
- extremely concerned (5)

Q30 Who do you feel most frequently initiates a conversation about Canine Cognitive Dysfunction Syndrome in a patient at your practice?

- the veterinarian (1)
- the veterinary technician/nurse (2)
- the client (3)
- depends (please explain) (4) __________________________________________________
- other (please explain) (5) __________________________________________________
- I have never had a conversation about Canine Cognitive Dysfunction Syndrome in my practice (6)

Q31 What resources related to cognitive dysfunction do you provide at your practice for clients? (click all that apply)

- client handout (1)
- website (2)
- primary literature (peer reviewed article) (3)
- support group contact (4)
- other (please explain) (5) __________________________________________________
- none (6)

Q32 In your experience, what is the primary reason most owners elect humane euthanasia for their dog diagnosed with Canine Cognitive Dysfunction Syndrome?

- disorientation (1)
- changes in social interactions (2)
- changes in sleep/wake cycles (3)
- increased house soiling (urination or defecation) (4)
- anxiety (5)
- aggression (6)
- a combination of these (7)
- other (8) __________________________________________________
- I have never had a patient be euthanized who was diagnosed with Canine Cognitive Dysfunction Syndrome (9)

Q33 Have any of your clients/patients participated in a clinical trial for Canine Cognitive Dysfunction?

- yes (1)
- no (2)

Display this question:

If Have any of your clients/patients participated in a clinical trial for Canine Cognitive Dysfunction? = yes

Q33A Please provide the name of the trial or a description and location/sponsor of the trial (if known).

________________________________________________________________

Q34 Would you be interested in hearing more about Canine Cognitive Dysfunction and related clinical trials?

- yes (1)
- no (2)

Display this question:

If Would you be interested in hearing more about Canine Cognitive Dysfunction and related clinical t... = yes

Q34A What format would you like to receive this additional information regarding Canine Cognitive Dysfunction and related clinical trials?

- clinical trial website (1)
- continuing education (2)
- through your VMA (3)
- flyer/direct mail (4)
- email (5)
- radio/website advertising (6)
- Facebook/Instagram/TikTok (7)
- other (please explain) (8) __________________________________________________

End of Block: Senior Pet Clients
